# Acute ischaemia of the upper limb following peripherally inserted central catheter—a venous to arterial complication cascade

**DOI:** 10.1093/jscr/rjab188

**Published:** 2021-07-09

**Authors:** Khaleel A Hamdulay, Rene van den Bosch

**Affiliations:** Department of Vascular, Endovascular and Transplant Surgery, Christchurch Hospital, Canterbury District Health Board, Christchurch, New Zealand; Department of General Surgery, Timaru Hospital, Southern Canterbury District Health Board, Timaru, New Zealand

## Abstract

Peripherally inserted central catheters (PICC) are useful access devices that allow for longer-term intravenous access. This allows patients requiring an extended period of intravenous medication to have this administered without the need for repeat vascular punctures. Even minimally invasive procedures such as line insertion come with risks. Of particular interest to this article is a limb threatening complication soon after line placement. We discuss the PICC line catheter tip as the likely cause for arrhythmia that lead to an embolic occlusion of an upper limb and required acute surgical intervention for limb salvage. We stress the rapid sequence of events that lead to this ultimate complication. We also stress the importance for all clinicians to be aware of these risks and take a cautious approach as the majority of patients requiring longer-term access are already at greater risks of thromboembolic disease due to their comorbidities.

## INTRODUCTION

Peripherally inserted central catheter (PICC) lines are commonly used for patients requiring medium- to long-term intravenous access [1]. Reasons include for administration of intravenous antibiotics or chemotherapy. Placement of PICCs comes with risks, including arrhythmias from suboptimal positioning. Although arrhythmias have been documented, there is no literature of related acute sequelae leading to acute surgical intervention being necessary.

We discuss an interesting case in which acute limb ischaemia arose as a result of arrhythmia caused by malpositioned PICC insertion. Of particular interest is the rapid onset of the complication and the immediate treatment required for limb salvage.

We stress the importance of realizing the true risks involved with these procedures and the cascade of potential resulting complications that may follow. A single complication may be further complicated with another. The complication cascade may be both rapid and delayed in onset.

## CASE REPORT

We discuss a 59-year-old female who presented for planned PICC insertion for chemotherapy administration. Her medical history consisted of sigmoid adenocarcinoma with a Hartmann’s procedure the month before and subsequently commencing chemotherapy a week before. She was a non-smoker and had no familial history of coagulation disorders.

**Figure 1:**
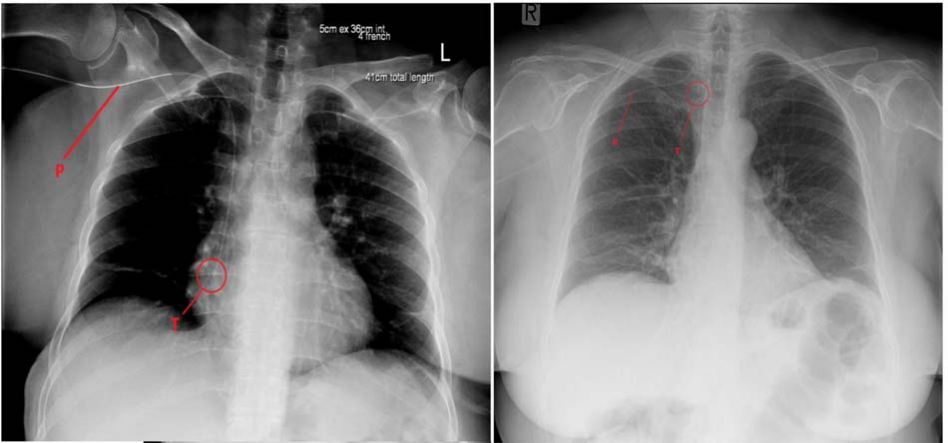
Chest X-ray after (left) PICC insertion and after withdrawal (right). P—PICC line tubing traced to the tip (T) in the right atrium (left) and superior vena cava (right).

The PICC line was inserted into the right arm under ultrasound guidance, without any immediate concerns noted during the procedure. An X-ray revealed the PICC tip to be in a satisfactory position, but potentially a little too deep (Fig. 1). There were no symptoms of chest discomfort or palpitations.

**Figure 2:**
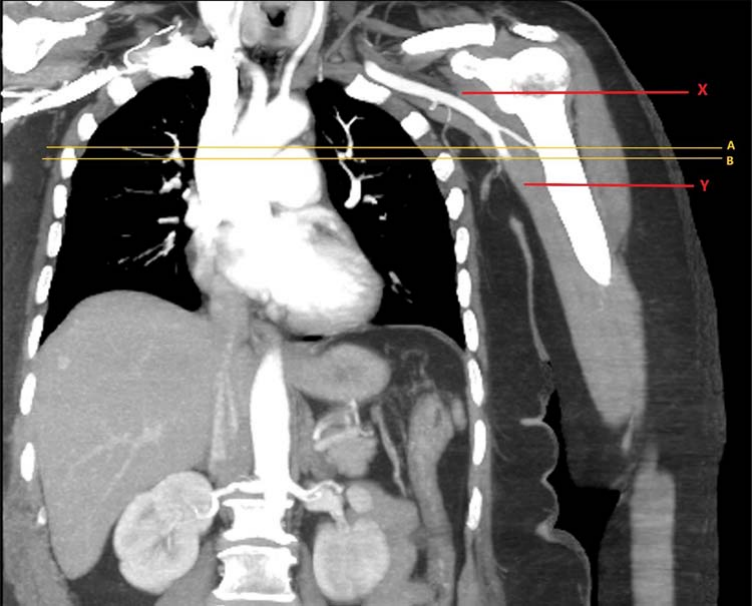
CTA of left upper limb in the coronal plane. X—axillary artery with contrast showing flow as evident in transverse imaging (see Fig. 3), patent in the transverse plane marked A. Flow ceases approximately at the level of the plane marked B. Y—axillary artery occluded as shown with contrast absent.

**Figure 3:**
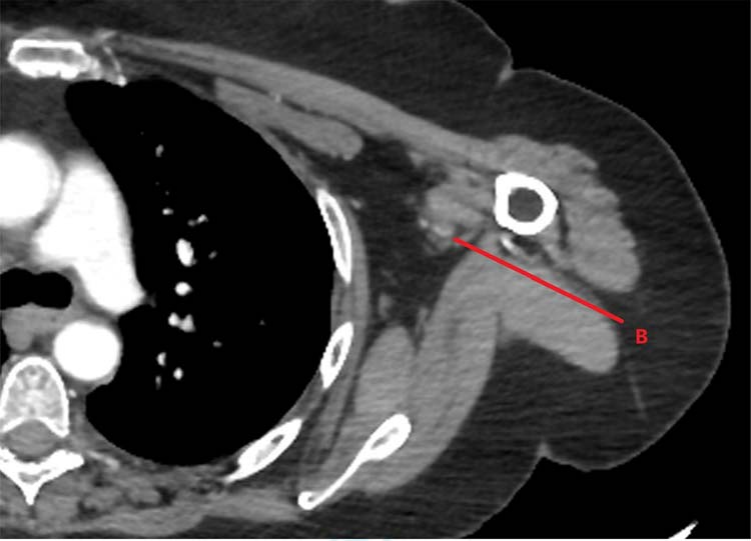
CTA of left upper limb in the transverse plane. B—axillary artery without contrast showing occlusion due to embolism as evident in transverse imaging at B-line (see Fig. 2).

Approximately an hour later, she developed symptoms of pain and weakness to her left arm (the opposite side). Her arm was clinically assessed as cool to touch with a delayed capillary refill with pulses absent. A computed tomography angiogram (CTA) of her left arm was arranged which revealed an abrupt stop in the left axillary artery (Figs 2 and 3) with reconstitution of the vessels in the antecubital fossa. She was taken to theatre for a brachial embolectomy to re-establish blood flow to her acutely ischaemic arm.

Brachial access was obtained over the antecubital fossa, the brachial artery was controlled and incised. Proximal embolectomy with balloon catheter retrieved a long segment of thrombus, with resulting good inflow and back bleeding. Post-operatively she was commenced on therapeutic low molecular weight heparin (LMWH).

She was subsequently worked up for a cause for this limb threatening pathology. Echocardiogram revealed unremarkable cardiac valves without evidence of an embolic source. A bubble study was also performed which showed no evidence of right to left shunt or patent foramen ovale. Histology confirmed recent blood clot without organization. At follow up she had a good radial pulse and her LMWH was switched to rivaroxaban.

PICC lines are generally considered safe, with delayed complications including occlusion, accidental withdrawal, infection and thrombotic complications [1]. Immediate complications are well documented, including those of a cardiac nature due to the tip in contact with the right atrium; atrial or ventricular contractions leading to atrial fibrillation (AF), supraventricular tachycardia or cardiac arrest [2].

It has been reported that PICC line insertion can affect the right atrium and result in AF, and cease when the PICC tip is retracted [3, 4]. X-ray has been suggested as the gold standard for post procedural check, but this only gives an anatomical estimate of positioning rather than functional—it has therefore been suggested that electrocardiogram (ECG)-assisted placement is performed to assess for arrhythmias such as AF [5]. Stroke is the most widely documented risk of AF with the left atrial appendage also being affected and therefore allowing for embolic propagation from the left heart into the arterial circulation [6]. It should however be noted that other arterial embolization, although only accounting for <10–12% of events, can be just as devastating [7, 8].

Our case suggests a subclinical episode of AF from PICC line insertion which resulted development of thrombus. This propagated to the left arm causing an acutely ischaemic limb. The rapid succession of events leading to this is of most interest. Of course there are pre-existing risk factors in our patient that predisposes her to being hypercoagulable (recent surgery and cancer diagnosis). It is likely that these factors all contributed to her overall hypercoagulable status, in addition to the likely arrhythmia acting as a trigger. Unfortunately no post procedural ECG was obtained to confirm AF and our suggestion is of course speculative. No other cause was found on investigatory work-up.

Right arm venous access resulting in left arm arterial embolism can only be explained by an event affecting both cardiac atria. The right arm sustained no complications and working PICC line would confirm correct entry along the venous system. The possibility of right to left shunt was excluded on investigation as this would cause embolism propagation from the left atrium to the arterial system. Without a physical defect electrical conduction across the cardiac septum is a possible explanation. It is known that an electric connection exists, starting with the PICC triggering arrhythmia in the right atrium and electrical conduction propagating from there to the left atrium via the coronary sinus musculature connection [9]. Although arrhythmias can communicate between atria, this eliminates the need for a thrombus to develop in the right atria and travel through the lung filter. It is more likely that the thrombus origin is in the left atrium.

## CONCLUSION

Thrombotic and embolic events are known to occur in high-risk individuals with comorbidities that predispose to hypercoagulable states. In order to help these patients with treatment we sometimes have no choice but to subject them to procedures that carry the risk of triggering events that lead to life or limb threatening complications. Although this is not a reason to be risk averse, clinicians need to be aware of these complications arising any time from an immediate to delayed timeframe. One complication may lead to another and the possibility of this needs to be considered. Informed consent should of course be taken and emphasis placed on patients returning if any untoward effects develop after the procedure. This would warrant prompt investigation and identification of the complication so that timely intervention can take place.
